# Perceived Impacts of Health Care System Navigation on Health and Well-Being Among Patients With Musculoskeletal Conditions: A Qualitative Study

**DOI:** 10.1177/11786329261475438

**Published:** 2026-08-03

**Authors:** Paige Campbell, Geneviève Jessiman-Perreault, Jean Miller, Romita Choudhury, Kyle McCallum, Jason Martyn, Mel Slomp, Jason Werle, Jill Robert, Breda Eubank, Ania Kania-Richmond

**Affiliations:** 1 Cumming School of Medicine, 70401 University of Calgary, Calgary, AB, Canada; 2 3146 Alberta Health Services, Edmonton, AB, Canada; 3 Faculty of Humanities and Social Studies, 1349 Athabasca University, Athabasca, AB, Canada; 4 Faculty of Kinesiology and Applied Health, 8665 University of Winnipeg, Winnipeg, MB, Canada; 5 Department of Surgery, Cumming School of Medicine, 70401 University of Calgary, Calgary, AB, Canada; 6 Institute for Improved Health Outcomes, Calgary, AB, Canada; 7 Primary Care Alberta, Calgary, AB, Canada; 8 Health & Physical Education Department, Faculty of Health, Community, & Education, 2123 Mount Royal University, Calgary, AB, Canada; 9 Department of Community Health Sciences, Cumming School of Medicine, 70401 University of Calgary, Calgary, AB, Canada

**Keywords:** Canada, care access, health care delivery, health outcomes, health system navigation, musculoskeletal disorders, patient experience, qualitative research

## Abstract

**Background:**

Musculoskeletal (MSK) disorders are a leading cause of disability and health care burden globally. While much research has focused on clinical outcomes, treatment efficacy, and system efficiency, less is known about how challenges in navigating the health care system affect patients’ physical, psychological, and social well-being. Understanding these lived experiences is crucial to informing system-level care improvements.

**Objective:**

To explore patients’ experiences navigating the health care system and the associated impacts on their physical, psychological, and social well-being among individuals with musculoskeletal conditions in Alberta, Canada.

**Design:**

Qualitative study using the interpretive description approach.

**Methods:**

Semi-structured interviews were conducted by telephone with a purposive sample of adult participants who received care for knee (soft tissue), low back (spine), and/or shoulder conditions. Data were analyzed using a framework analytic approach, with particular attention to themes derived from the data related to negative health impacts associated with system navigation.

**Results:**

Seventy-three participants completed an interview. The majority of participants described negative health impacts, which they attributed to challenges navigating the MSK health care system. Patient accounts reveal a spectrum of negative health impacts, including physical health deterioration over and above the MSK condition, a range of mental health challenges, and a decline in social well-being. Participants linked these experiences to specific system-level factors such as delays in receiving care, inconsistency in communications and treatment recommendations, care fragmentation, and affordability barriers.

**Conclusions:**

These findings underscore the challenges of navigating the health system with an MSK condition, which can hinder recovery, exacerbate suffering, and add additional health burdens. Greater attention to the lived experience of system navigation is needed, alongside integrated, timely, person-centred and holistic approaches to MSK care.

## Introduction

Musculoskeletal (MSK) disorders are a leading cause of disability in Canada and globally, accounting for a large burden of disease, reduced quality of life, and high health care utilization.^1-3^ Conditions such as osteoarthritis, low back pain, and neck and shoulder dysfunction are among the most commonly reported issues in primary care.^4-6^ Despite the high prevalence of these conditions, access to timely and effective MSK care in the Canadian health care system remains fragmented. System-level challenges, including long wait times, shortage of family physicians, siloed services, and inadequate funding for allied health professionals, have been consistently reported across multiple Canadian jurisdictions.^7-9^ These issues are particularly prominent for MSK patients, who often navigate a complex web of generalist and specialist providers, diagnostic delays, and restrictive referral pathways.

There is an increasing emphasis in health services research to better understand the patient experience as a means to improving care quality and advancing a patient centered approach in the delivery of health care services from physicians (family physicians, sports medicine physicians, orthopedic surgeons), rehabilitation professionals, (physiotherapy, athletic therapy, chiropractors, massage therapists, etc.).^10-12^ Previous studies have explored dissatisfaction of MSK patients with access, provider communication, and service coordination.^10,13-19^ Importantly, the process of navigating the health care system itself influences not only patient perceptions of care quality but also their health outcomes. While many MSK conditions are chronic and may deteriorate regardless of intervention, delays in care, inconsistent treatment recommendations, and repeated episodes of ineffective management may not only preclude recovery but also compound physical dysfunction, chronic pain, or emotional distress.^10-12^ However, the holistic health impacts of navigating the health care system remain poorly understood.

Holistic health offers a broader understanding of health, taking into consideration the interconnection between physical, mental, emotional, social, and financial well-being.^17,20^ Within the context of MSK care, this perspective is particularly relevant as it emphasizes that health extends beyond the physical disease and its management alone. A holistic health lens draws attention to the fact that MSK conditions, which are primarily physical, impact other dimensions of health, which may also require intervention. Understanding the holistic health impacts of system navigation, therefore, provides important insight into how system structures and processes compound the burden of MSK conditions and more accurately reflect the full patient journey through the MSK system.

This research is focused on the human experience of living with the broader health consequences that arise as a result of the challenges encountered when attempting to seek health care services and navigate the health care system. This study aimed to understand how patients with knee, shoulder, and low back (spine) conditions in Alberta, Canada, perceived the impact of navigating the public health care system on their musculoskeletal health. Using a large qualitative dataset, we examined the physical, psychological, and social effects on patients as they moved through their MSK care journey. These patient narratives illuminate not only the gaps in care coordination and service delivery but also their health impacts that are often not evident in administrative data or clinical outcomes.^21,22^

## Methods

### Study Design

This study employed an interpretive description approach, as outlined by Thorne et al,^21^ specifically developed to generate experience-based insights for direct application to care delivery and program implementation. Reporting in this paper adheres to the Consolidated Criteria for Reporting Qualitative Research (COREQ)^23^; see supplemental file 1. Ethical approval was granted by the University of Calgary Conjoint Health Research Ethics Board (REB22-0881).

### Study Setting

The study took place in Alberta, the fourth-largest province in Canada, which has a population of 4.5 million people. Approximately half the population resides in the metropolitan centers of Calgary and Edmonton.^24^ Alberta’s health care system provides publicly funded access to medically necessary hospital and physician services, as well as select rehabilitation services. Most allied (e.g. physiotherapy, occupational therapy) and complementary services (e.g., massage, chiropractic treatments, acupuncture) are not included under public funding and are paid for out-of-pocket by the patient or their expanded insurance coverage.^25^

### Sampling and Eligibility

We used purposive sampling with maximum variation to capture a broad range of experiences across age groups, sex, musculoskeletal (MSK) conditions (knee, shoulder, low back), geographic regions, and care pathways. Inclusion criteria were adults (≥18 years) residing in Alberta who had received or tried to receive health care services for a knee, shoulder, or low back MSK condition. Exclusion criteria included inability to participate in an English-language interview, cancer diagnosis affecting the bones of the knee, shoulder or low back, and/or were on the surgical care path for knee arthritis.

### Participant Recruitment

We employed a maximum variation and stratified sampling strategy to constitute a sample of adult individuals with MSK conditions. Recruitment was multi-pronged using online and in-person strategies, and data was collected from November 2022 to December 2023.

### Sample Size

Consistent with principles of qualitative sufficiency and thematic saturation, we continued with interviews until no substantively new insights or themes were identified during our on-going analysis and inclusion from across defined geographic regions and joint conditions was achieved.^21,26^

### Data Collection

Individual interviews were conducted by telephone, in a location selected by the participant (most often at home), and lasted between 40-70 minutes (conducted by: AKR, KG, JM, KM, PC). Interviewers introduced themselves as research team members, and their role(s) in relation to the project. There were no prior relationships with study participants. A semi-structured interview guide was used (Supplementary file 2); wording and question sequence were iteratively modified based on preliminary analyses. With the exception of one interview that was repeated due to a recording failure, all participants completed one interview. Verbal informed consent was obtained because interviews were conducted by telephone, making written consent less feasible and potentially burdensome for participants. This consent process was approved by the University of Calgary Conjoint Health Research Ethics Board, and verbal consent was documented at the start of each interview within the corresponding study records/audio-recordings prior to data collection. Verbal informed consent was obtained at the beginning of each interview and documented within the corresponding files (audio-recordings, transcripts). Participants were provided with study information in advance, and consent included agreement to recording, transcription, and use of de-identified quotations in the presentation of findings. The consent form was modified to include mental health resources with the research team’s recognition that the interviews were eliciting recall of situations in their experience of the health care system that were upsetting to participants. Data collection continued until data saturation was reached, whereby no new themes or insights were identified.^26^ No participants withdrew from the study.

### Data Analysis

All interviews were audio-recorded, de-identified, and transcribed verbatim. NVIVO 14 software with the collaboration cloud was used to facilitate data management and analysis.^27^ Analysis followed a framework analysis approach.^28^ A focused secondary analysis was conducted by two members of the analytic team (AKR, PC) specifically on the theme of negative health impacts from system navigation, derived from the data during the first round of the analytic process. Member checking was completed with a sub-sample of 19 volunteer participants,^29^ confirming the interpretation and presentation of the findings.

### Researcher Reflexivity

Interviews were conducted by a research team (AKR, KG, JM, KM, PC), whose members had PhD-level training (AKR, KG, JM, PC) and other graduate training. At the time of the study, members were medical trainees (PC), qualitative researchers (AKR, KG, JM, KM), patient partners (JM, RC) and civil servants (AKR, KM). Members identified as female (AKR, KG, JM, PC) and male (KM). Interviewers had prior experience in qualitative research or received training in semi-structured interviewing and qualitative methods. The multidisciplinary composition of the research team supported reflexive and in-depth data analysis. Reflexive practices were employed throughout, including bracketing, memoing, and weekly team discussions to examine emerging interpretations and consider how researchers’ perspectives may influence findings.

## Results

Seventy-three participants were interviewed between November 2022 and December 2023. The median age of participants was 47.5 years (range: 20-79 years). There was a higher proportion of participants who identified as women (62%). Participant characteristics are presented in Table 1, including distribution across joint conditions: low back (spine) (27%), knee (soft tissue) (27%), shoulder (15%), and multiple joints (30%). Participants sought services from a range of health care professionals, and most required non-operative pain and dysfunction management rather than surgical intervention.

**Table 1.** Study Sample Characteristics (n=73)

Patient characteristic	Median (IQR)
**Age**	47.5 (26.3)
​	**Percent (n)**
**Sex**	
Women	61.6 (45)
Men	38.4 (28)
**Geographic location** *	
North Zone	6.8 (5)
Edmonton Zone	21.9 (16)
Central Zone	4.1 (3)
Calgary Zone	56.2 (41)
South Zone	11.0 (8)
**Joint of Concern**	
Knee(s)	27.4 (20)
Shoulder(s)	15.1 (11)
Low back	27.4 (20)
Multiple joints (combination of knee, shoulder and/or low back)	30.1 (22)

*At the time of this research, the province of Alberta was divided into five administrative health zones: North, Edmonton, Central, Calgary, and South [36].

Negative health impacts as a result of health system navigation were elucidated by more than half of the participants (60%); this reflects the proportion of participants whose narratives included descriptions of negative health impacts which they attributed to system navigation. Three themes were constructed during the analytic process and reflect negative health impacts, over and above the MSK condition, attributed by participants to system factors: 1) physical health deterioration; 2) mental health challenges; and 3) decline in social well-being. The system factors associated with these negative health impacts, as identified by participants, occurred at various stages of their care journeys (diagnosis, treatment, follow up, and continued care), and included waiting for care, disconnected services, lack of providers’ MSK knowledge, lack of access to treatment, ineffective treatments, lack of compassionate care, and out-of-pocket costs. Table 2 describes each factor and presents illustrative quotes.

**Table 2.** Health System Causes Associated With Negative Health Impacts

Cause	Description	Illustrative quotes
Waiting for care	Participants consistently experienced prolonged wait times for or delay in health care services including treatments, diagnostic imaging, assessments, specialist consultations, surgery. Often the waiting occurred without any information about a potential window or date for the required intervention.	Pa 1001: “*I just couldn’t get any where. I couldn’t get my family doctor… like it took a long time to get him to make that referral to the spine clinic. He just felt that there were other things we should do first. And I suspect, in the medical world of Alberta, they really don’t want you, I mean they probably realize how few neurosurgeons and spine clinics there are in Alberta. So, before we make any referrals, let’s do all the things we can do, right? But—sorry, …everything took so long. All those things (appointments at clinics, with specialists) took weeks or months to arrange before I’d have the next appointment, right? So, I was just getting to the point where I’d be walking half a block and I felt like I’d gone 10 miles, oh my goodness.... there’s got to be another way”.*
		Pa 1017: *“…at this point, after three years, it’s not getting better with physiotherapy, chiropractic therapy, massage therapy. Like nothing is helping. So, I had gone back to him [family doctor] and I was like, we need to do something here. Like what are my options? So, he finally sent me to a surgeon...the surgeon is going to send me for, to this specific shoulder specialist. But he’s, he’s so good he said, that the appointment that I get with him is probably not going to be for like six months to a year. And I’ve already been suffering with this for three years.”*
Disconnected services	Participants experienced disruptions in their care resulting from a lack of clear, consistent communication and poor coordination between providers or clinics. This resulted in lack of clear direction, follow-up care, and lost referrals.	Pa 1016: *“I was just didn’t have very good discharge information. My father-in-law’s a surgeon and wrote a letter to the unit because he was like, this discharge was so poorly done. He was there for it. And I was just rushed and there was like no good info—specific information. And I had the wrong follow up information.”.*
		Pa1081 *“I ask: how do I book my next follow up appointment with Dr. Jones? One staffer says, oh, you have to go back to the front desk there and talk to this lady and she’ll book you. Another says, oh, we’ll call you, and we’ll let you know when you have to come in. There was not consistency with that. So, there’s times I go to the front desk, and they would say, no, no, we’ll call you. And then nothing would happen. I’d call and they say, oh, yeah, you have to book it. Well, you’re giving me two completely different things here. I don’t understand why rebooking people after surgery should be confusing for the patient”.*
		1072: *….Scattered information. Lack of communication between health care providers. I give so many consents. Everywhere I go I find a piece of paper saying that I give my consent to share this, to share that. And I understand why. But if I give them the consent to do this or that, I can also give them the consent to share the findings about my health with other health care providers…And another word would be duplication. Duplication of efforts. Duplication you know, spending so much money on doing the same kind of tests over and over again...* *I mean everyone is willing to help, that’s a plus.*
Lack of MSK competence	Participants encountered situations where providers lacked the necessary skills and knowledge to effectively address their MSK problem. This was experienced as conflicting information from different providers, incomplete or inadequate assessments, resulting in incorrect assessments, and inappropriate referrals.	Pa 1039: *“… everybody keeps telling you, ‘Oh, it’s just muscle, don’t worry about it. You know, you’ve lived this long, blah, blah, blah. Everybody will have these kinds of back things. Don’t worry with an MRI because the MRI’s just going to show what everybody else your age would likely have in their lumbar spine’. So, I went in to see this one physiotherapist …and he gave me the most thorough assessment I’d ever had. Family doctors, the orthopedic surgeon—nobody had done an assessment like this…what I found with both family doctors, is they didn’t actually physically examine me. They just listened to my history. And then they made a judgement. And he said, ‘oh my God, you are urgent! You need to get into [speciality back clinic name]. And so, he wrote three letters and made several phone calls. And finally, I got an appointment in the [name of clinic].”*
		Pa 1076: *“The surgeon looked at it and said, this should have been operated on immediately. It would have been a perfect candidate. But now I would have to scrape all sorts of growth, and I’d have to go in and it’s gone from a very simple operation to a very complex operation, which has high risk. I listened to the second surgeon, and he told me that like, you know, live with it. And the first surgeon is the one who told me I could go to physio, which I did. Frustrating, obviously. You could tell just from the fact that I’ve talked about the fact that one doctor told me one thing, another doctor told me another thing. And bluntly, the two doctors told me two entirely different things. One is saying, let’s give it a little bit of time, and it doesn’t matter. And the other is saying, this should have been fixed on day one, and now it does matter.”*
Ineffective care	Participants expressed that treatment or recommendations from their health care providers did not produce results that led to resolution of their MSK problem or condition or effective management of a long-standing condition. Many felt that their providers offered only temporary solutions or had little to offer in terms of effective treatments or solutions for their conditions.	*Pa1044: the individuals I dealt with were all very compassionate, but they just had nothing to give me. They, they had no, no access to whatever it was I needed at the time. And I, you know, in retrospect what I expected—what should have happened is you know, we should be doing disc replacement surgery here. And there should be access to it. And neither of those things exist unfortunately.*
		*1037:* *I’ve already lost 11 years...having discussions—what feels like we’re just having discussions—and no real action is being taken to resolve the issue. I mean, I could have my quality of life back. I could have had it back 10 years ago. In the States. I would go down there and get the procedure done. unfortunately, some of these procedures are not offered in Canada…. But it just doesn’t seem like, for lack of a better term, rocket science to offer disc replacement in Canada.*
		Pa 1078: *“Part of the frustration of this is that all the exercises I was given, and I did them, and I wasn’t really improving any more. Right? So, it’s like, it still continues to be a challenge for me to go down the stairs and to run. Like all these things and the winter.”*
Lack of access	Participants experienced an inability to access or obtain needed health care services resulting from unaffordability and lack of public funding for needed services, and services not being available in one’s home jurisdiction.	Pa 1043: *“...and especially the mental health aspect of it. Like, telling my family doctor, hey, I am feeling really depressed. This (MSK issue) is really hard on me. I am having suicidal ideations. I don’t know where I’m going with my life. And she was like, well, there is this cognitive behavioural therapy program... and I’m like, okay I can commit to a month of doing that. Well, oh, there’s a waiting list. And it’s six months or it’s eight months... And so, I’ve started the process to do that and then somehow nobody’s ever followed up with me on it…. I’ve gone in, done the initial intake, and then I never get called back. I never get help because I’m sure there’s so many more people that are walking into Emerg and saying I’m going to kill myself right now. Those are obviously the people that get triaged and go to the top of the list. I’m somebody that, you know, I have supports and family and friends and things like that. I manage.... I’m trying to do things myself and cope the best that I can on my own, but that piece, for sure, has been a huge frustration. And mostly because it’s like, oh well, it’s not acute enough.”*
		Pa 1047: *“…my dad actually had a similar problem, and he went to private care in British Columbia. And so, he suggested I go, and it was just eye-opening…they asked ‘like you—why have you never had a full body x-ray? Why haven’t you…’ And by this time, I just couldn’t do anything, and it came to the point where the back was so bad, like I, I was starting to lose feeling in my legs, certainly in my feet quite often. And my bladder was starting to be affected, so it was pretty—pretty serious by the time I got help. And I was on a lot of T3s, like over the course of that last six months before my surgery, I was maxed out. … But the week after my surgery (at a private clinic in BC), when I walked out, and never had another painkiller. And I walked out. Like, amazing difference. Even the ride home was better because it was all the way to Calgary. And the ride there was super horrible, and the ride back was better. The lack of pain is huge.”*
Lack of compassionate care	Patients perceive a lack of empathy, listening, or understanding in the approach used by providers when addressing patients. Many participants expressed that their concerns were dismissed, ignored, reduced or addressed in a flippant manner by health care providers. They felt unheard and not taken seriously, resulting in delayed diagnoses, inadequate treatments, and growing frustration.	Pa1016 “…the pain was like so bad that I was like basically, I wasn’t even crying. I was just like having a pain crisis. And um, I asked for something, and the nurse was like, no, we don’t have time. And she just left me in the wheelchair and was like you’re just going to have to go physio as is. Um so I went down, and I was like, okay, maybe I’m just not being tough enough. And then the physio brought me back, right back up, and was like, there’s no way you can do it with this amount of pain. And so that delayed my physio”
		Pa 1053: *“My family doctor kind of sort of brushed it off a little bit. He kind of was like, oh, it’s not too bad. Like I’ve seen people that are way worse than this. I’ll give you some muscle relaxants and anti-inflammatories and couple of weeks you’ll be okay, kind of thing. I’m like, oh, okay, maybe that, maybe it’s not too bad. Maybe I just need to stretch it out, or you know, go to physio a bit and do that. But you know, it turned out to be not the case....and like I just wasn’t really getting anywhere with, with that line of treatment.”*
		Pa 1087: “*I think there’s something with, when you’ve had a condition for a long time, that there’s almost a dismissive nature about it. Like the longer time goes on, the less the system prioritizes it.”*
Cost of health care	Patients assume a high financial burden for health care services due to the lack of services available within the public system or needing to seek care out of province or out of the country.	Pa 1044: *“…the options available to me through the public health care system in Alberta just weren’t an option. Between wait times and just the fact of the surgery I needed was not available here (in Alberta) drove me to get health care elsewhere at considerable cost.”*
		Pa 1048: *“I had a series of injections into my facet joints as well as a nerve oblation. and physiotherapy… But I can’t really say that those treatments did anything because as I said, my need for (on-going) physiotherapy and massage and what not, is pretty consistent. But the past couple of years, it’s gone just bonkers like with what I’m spending out of pocket…”*

### Physical Health Deterioration

Many participants, who were entering the system in an already vulnerable state with debilitating pain and mobility impairments, experienced further physical decline due to worsening or unresolved symptoms from their original MSK injury. Participants felt these issues were caused by the system itself being a barrier to managing their health. More specifically, participants spoke of a lack of perceived urgency regarding their MSK issues, which they felt further exacerbated their inability to access health care services in a timely way.

#### Worsening or Unresolved Symptoms

Participants experienced a physical decline such as increasingly reduced mobility and function, perceived ongoing damage to the physical structures (e.g. joint and muscle tissues), and higher pain levels. For some, this resulted in significant changes to their physical abilities: “*I can’t walk more than a hundred steps . . . two aisles of a grocery store”* (Pa 1014). The decline often occurred over several months, and sometimes years as they waited for access to imaging, specialist appointments, and/or treatment.

*“I just couldn’t get anywhere. I couldn’t get my family doctor… like it took a long time to get him to make that referral to the spine clinic. He just felt that there were other things we should do first. And I suspect, in the medical world of Alberta…they probably realize how few neurosurgeons and spine clinics there are in Alberta. So, before we make any referrals, let’s do all the things we can do, right? But—sorry, …everything took so long. All those things (appointments at clinics, with specialists) took weeks or months to arrange before I’d have the next appointment, right? So, I was just getting to the point where I’d be walking half a block and I felt like I’d gone 10 miles, oh my goodness.... there’s got to be another way”. (Pa 1001)*

#### Permanent Damage

For some, the physical deterioration resulting from waiting, ineffective care, and circling the system looking for help left them in a place where “*the damage is done*” (Pa 1023), with the initial problem transitioning into a chronic lifelong problem:

*“…then it was well over a year before I could even get the MRI. I waited a long time for that. And then they said, it’s (the shoulder) is completely torn. You need to see a surgeon. I did see a surgeon...and he looked at the MRI and went, oh yeah, absolutely, its fully torn. He goes, now it’s been a little bit too long, wish we would have done this sooner. And I said, well, it was 11 months ago that I had the MRI, and I waited over a year for it, almost a year and a half. So, I asked, you know, what can I do? Like, I got, I got a little guy at home. I got to be able to you know, do stuff.... with my shoulder - nothing that they tell me that they can do. And really, I’m just left with a bum shoulder. And that’s it”. (Pa1015)*

#### Development of Co-morbidities

Some participants described the development of new health problems, such as altered biomechanics, limited function or mobility, high fatigue, poor sleep, weight gain and chronic pain. Overall physical decline was a pervasive theme and new pain symptoms often emerged.

*“…waiting those seven months… then other things are going wrong. Now I’ve got nerve pain down my leg. Now that nerve is atrophying. Now there’s other issues. Now I can’t bend my toe. So then when I finally do have the surgery, I’ve got a list of other issues that have, you know—or if my knee is in bad shape, then all of a sudden, I’m walking with a limp. Now my back is in bad shape too.” (Pa 1014)*

*I think the, the greatest challenge is when it’s not an emergency, it’s—the wait times, as I said, 9, 10 months for an MRI. And what I’ve also found is that other problems can set in that if you’d been able to get health care in a more timely manner, you wouldn’t have had the extra problems. Like the inflammation that sets in and becomes chronic... I have to live with them. And you know, I can’t tell you that, that if somebody had seen me right away, I wouldn’t have had any problems with them. But they tend to compound because you’re waiting. So, you’re waiting with an injury. (Pa1065)*

Participants noted the lack of opportunities to address these issues and concerns during clinical encounters. The restricted time during an appointment and limit of one main issue per visit created a narrow focus whereby providers did not allow for these resulting issues, deeply intertwined with the initial MSK condition, to be accounted for; thus impeding recovery and diminishing patients’ sense of support.

### Mental Health Challenges

Beyond the physical toll on participants, the journey through the health care system was often emotional, and at times traumatic, deeply affecting some participants.

*Interviewer: Were you satisfied with your care?*

*Participant: No [shaky laugh]. Sorry, I, I just, it really affects me. It’s, it’s been an emotional journey [voice breaks]. (Pa 1037)*

#### Psychological Distress

Anxiety and stress were a consistent experience associated with lack of clarity from the health system regarding their health issue and next steps. Not knowing what was wrong or what lay ahead was often cited as being almost as debilitating as the physical symptoms and pain itself.

*“The uncertainty about what’s going on is what weighs on me the most…Living in limbo—knowing something’s wrong but not having answers—is exhausting.” (P130)*

There were several other factors that contributed to the mental burden associated with health care system. Participants expressed frustration with the lack of access to effective treatment options and concerns about being prescribed medications, specifically opioids, given the potential of addiction.

*I’m unemployed (unable to work due to health problems). I can’t walk. I can’t lift. I can’t sleep. I’m on medication that makes me so drowsy I can barely formulate an email to, so that I can like at least function as a human being getting my shoulders (looked after somehow). So, like —I’m terrified. What, like, what are my next steps? My next step is I go for a massage tomorrow morning. Like, right, like that’s not putting my mind at ease. You know? Sorry, I’m just—it’s frustrating. It’s so frustrating. (Pa 1062)*

Costs associated with health care were a significant source of stress, particularly for those without third-party (private) insurance plans or the financial resources to pay privately for assessments or treatments. Anxiety and stress were further aggravated by a sense of being dismissed, ignored or inadequately listened to by health care providers, also leading to feelings of helplessness, disempowerment and frustration. For some, their health system experiences evoked depression.

*“I would say that I eventually did (receive needed care and advice, information), but the timeframe in which I felt I did not have enough information or knowledge or empowerment, was extremely difficult, extremely discouraging and depressing, honestly.” (Pa1039)*

The degree of psychological distress associated with anxiety, stress and depression indicated by participants’ experience ranged from mild and intermittent to severe, causing significant disturbances in participants’ mental health and for prolonged periods. For a few, the psychological toll was profound, with several participants describing significant mental health challenges, including suicidal thoughts, as they grappled with the inadequacies of the health care system, in addition to pain and dysfunction associated with their MSK issues.

*“If I was faced with 18 months to 24 more months of waiting and being in the condition I was in, I would not have survived that time. I would have died by [medication/drug] overdose, accidental or intentional…the life I was living was torture. And I, I know that sounds dramatic, but that is, I believe that with my whole heart.” (Pa1044)*

#### Loss of Self

The loss of a sense of self or self-identity was associated with the toll of navigating what was perceived as a complicated, unhelpful, and often dismissive health care system. Several participants described changes in their personality or lifestyle. To realize and accept the sharp decline from being able “*to do 30-kilometer hikes*” to feeling “*lucky if you can get around the block*” (Pa 1001) was a shock for participants. Many resigned from activities that drastically impacted their quality of life - “*I don’t play sports anymore, don’t ski anymore*” (Pa 1016*) and “I had to give up long distance fly fishing and volunteering” (Pa1049).* Participants expressed being left living lives that they did not recognize and could not come to grips with, compounding the emotional toll.

*“…I’ve literally been fighting for this (shoulder pain), this problem that makes it difficult for me to work at a desk. It makes it difficult to um, like I would never be able to play a musical instrument. Um I used to do, you know, cross stitch and things while I watched TV. There’s no way I can do that. When I drive, I can hardly drive long distances because I get this extraordinary pain. Anything that I do in front of my body. So, I, I describe all of this to him (a doctor) and he’s like, oh. He told me, this is one of the most common things that I do. This is one of the most common things that we see. And I’m like, what? I have been complaining to doctors about this for most of my adult life, and this is one of the most common things you, see? The most common treatments you give? Like I, I cannot believe I have lived with this all this time. When this is something that they see all the time.” (Pa1003)*

Participants’ experiences indicate there was often an unwillingness of their providers to “see” how their experience with the joint issue is inseparable from their person, their life, and need to regain a sense of normalcy. Too often providers did not go beyond *“…a 0 to 10 pain scale to ask how I feel right now, like how do I want to feel . . . how can we work together to get to that place? Is that place a reasonable goal?*” (Pa 1048). They did not “*take in any of the attitude, any of the emotion, any of the other stuff that hugely impacts a person’s pain level, recovery and all that*.” (Pa1015).

### Social Well-Being Decline

The effects of physical deterioration and psychological distress extended into participants’ relationships. The demands of poorly supported daily life with the MSK condition and persistent self-advocacy to gain access to needed services often resulted in social isolation. Physical limitations, persistent pain, impaired mental health and energy committed to navigating the health care system negatively impacted their ability to engage with family, friends and peers. Participants reflected on how they were no longer able to participate in family walks or bike rides, and volunteer activities. Their physical limitations also placed additional demands on partners, which strained relationships.

*Every few months I get a phone call, an automated phone call, saying we haven’t forgotten about you. But I haven’t seen anyone going ‘here, let’s do some pain management’ because I’m on a waitlist... And honestly, I’m just frustrated by the whole thing. But I’m waiting for the waitlist so that I can come in and be told that I’m too young—because everyone tells me that you have to be over 60 to get a knee replacement. And I’m 47 and I am constantly hearing the words, you’re too young. But in the meantime, my body is just crumpling around my knee, trying to support my knee. And I cannot play with my children [crying]…like, not being able to walk four—three blocks on a flat surface without severe pain…I can’t hike mountains. And that was a big part of what we did (as a family). So, I, I can’t do the walking. I can’t do the biking. It’s affecting my sleep. It’s affecting my romantic life. It, it affects everything. And it, cumulatively it’s also affecting my mental health. (Pa1066)*

Some had to resign from work, which not only reduced professional interactions with colleagues, but also introduced financial strain.

*“Yeah, and the financial issue. Like we went into debt because we’re down one income. And I, I don’t have any kind of health care benefits. So, paying for everything was out of my own pocket. As opposed to like having an insurance program that covered part of it and stuff. Once I was done work (unable to work due to unresolved health problems), that all went away. So that was another huge added stress. And we, we still haven’t paid off that debt. Like my husband, with that, he works ‘til nine o’clock every night. Yeah, still, to this day. So, it’s very challenging. Like it affects everything and everybody.” (Pa 1086)*

## Discussion

This qualitative study examined the experiences of patients with knee, shoulder, and/or low back (spine) conditions seeking health care services for MSK-related issues. A notable aspect was the extent to which patients perceived their inability to effectively navigate the MSK health care system as contributing to their worsening overall health. A spectrum of patient experiences emerged that encompassed holistic decline over and above the MSK condition, including physical health deterioration, varying levels of mental health and emotional challenges, a decline in social well-being, and accumulated financial burden. These tangible and far-reaching health consequences were related to prolonged uncertainty, inconsistent care, and barriers to accessing appropriate medical and allied health services and were identified by patients across all stages of their MSK journey. Participants’ accounts offer a nuanced perspective on the broader health care experience, revealing how the act of navigating the system itself can become a burdensome process, distinct from, yet compounding, the challenges of the MSK condition alone.

While some MSK conditions are inherently progressive despite access to evidence-based treatment, these findings suggest that system-level factors, such as prolonged wait times, poor communication, poor service coordination, and lack of access to expert-based MSK services, can negatively impact various dimensions of patients’ health as they manage their MSK condition. Although these findings do not aim to assess a causal relationship between system factors and health outcomes, they do provide evidence, through the lived experience, of how the process of seeking care can compound suffering, with participants perceiving these experiences as detrimental to their overall health and well-being.

Participants described psychological impacts, underpinned by frustration, stress, helplessness, and disempowerment, as being direct results of navigating care. These themes align with prior research documenting how barriers in access, ambiguity in care pathways, and perceived provider indifference can shape a patient’s sense of agency and coping capacity.^10,12,16^ Importantly, these findings also identified the siloed focus of services and primarily on the joint pathology, which failed to account for the broader health challenges experienced by participants, which were deeply intertwined with the MSK condition. Participants noted limited opportunities to address these concerns during clinical encounters, impeding recovery and diminishing patients’ sense of support. This resulted in a perception of navigating two parallel journeys – one visible to the MSK system and one invisible, where one is left to deal with these additional issues alone and suffer in silence.

These findings underscore the importance of incorporating the patient voice in the evaluation of current care models and system planning to ensure access, care continuity and person-centeredness. Qualitative inquiry reflects a lived knowledge base, which is less visible, if not invisible in administrative or clinical datasets, and yet equally relevant and important. As others have noted, qualitative research is a powerful tool for uncovering unseen impacts of care delivery design.^18,21,22^ As such, patient-reported experience enables us to identify areas of misalignment between system design and lived experience. These findings also highlight the unique needs of MSK patients, which are often chronic and multifaceted. This requires ongoing care and access to the services of various types of health professionals. Beyond physical treatments, MSK patients often need support in other health domains, such as mental health, nutrition and potentially social services. These health care needs require consideration in the context of a predominantly acute care- focused, physician-centered medical system.^14^

In this context, calls to action, such as those of Lewis et al. 2021,^11^ should focus on improving the experience of care delivery alongside clinical effectiveness and system efficiencies, particularly through integration and system navigation supports to enable better communication and coordination of services. This is particularly relevant in the context of chronic MSK conditions where early intervention, coordinated care and management, a holistic perspective, and relational, empathic care are essential.^10,15,17^ Examples of such initiatives in Alberta and other Canadian jurisdictions include centralized referral processes^30-33^ and team-based physician care models such as Rapid Access Clinics, which aim to provide patients with expert-level assessments, education, and ensure patients are on the appropriate treatment path.^34,35^

In considering these findings, it is important to acknowledge the strengths and limitations of this study. A key strength lies in the rigorous and in-depth qualitative analysis conducted, which included consensus-based coding, a secondary analysis, member checking, bracketing and memoing. The study was conducted by an experienced and diverse team that included patient-researchers; however, the analysis was conducted by individuals of the same sex, which may introduce a specific sex lens to the analysis. We used a broad, multi-pronged approach for recruitment; most recruitment occurred online. Limitations to this study may include a sampling bias whereby individuals who do not engage with the online platforms used for recruitment and have lower digital literacy are excluded. The interview guide was not pilot tested, which may have impacted the clarity and consistency of responses. Nonetheless, the guide was developed through an iterative process and underwent detailed review within the research team to support its content validity. Further, participation was voluntary and respondent-driven, potentially leading to self-selection bias of those with negative care experiences. Lastly, we engaged a large and diverse qualitative sample of participants (MSK conditions, sex, and geographic location). However, this study took place in a specific region (Alberta, Canada), which may limit transferability to other health care systems that differ in their regional health care policies and delivery of MSK care.

Future work should also evaluate interventions designed to reduce the burden of navigation, such as centralized referral pathways, rapid access clinics, integrated interdisciplinary care models, and embedded navigation supports, with attention to both clinical outcomes and patient-reported experiences. Expanding this research across diverse geographic, socioeconomic, and health system contexts would further strengthen understanding of how MSK care delivery can be redesigned to support more timely, coordinated, and person-centred care.

## Conclusion

These findings highlight challenges and impacts of navigating the health system with an MSK condition. These challenges often impede recovery or effective management of MSK conditions, while exacerbating suffering, undermining coping abilities, and burdening patients with additional health consequences. Findings call for greater attention to the lived experience of system navigation in health services research and prioritization of integrated models of care, timely access to MSK assessment and treatment, and a person-centred and holistic approach that facilitate provision of mental health and social supports. Addressing system navigation as a determinant of health may represent an important opportunity for improving patient outcomes in MSK care.

## Supplemental Material

Supplemental Material - Perceived Impacts of Health Care System Navigation on Health and Well-Being Among Patients With Musculoskeletal Conditions: A Qualitative Study Supplemental Material for Perceived Impacts of Health Care System Navigation on Health and Well-Being Among Patients With Musculoskeletal Conditions: A Qualitative Study by Paige Campbell, Geneviève Jessiman-Perreault, Jean Miller, Romita Choudhury, Kyle McCallum, Breda Eubank, Jason Martyn, Mel Slomp, Jason Werle, Jill Robert, Ania Kania-Richmond in Health Services Insights

Supplemental Material - Perceived Impacts of Health Care System Navigation on Health and Well-Being Among Patients With Musculoskeletal Conditions: A Qualitative Study Supplemental Material for Perceived Impacts of Health Care System Navigation on Health and Well-Being Among Patients With Musculoskeletal Conditions: A Qualitative Study by Paige Campbell, Geneviève Jessiman-Perreault, Jean Miller, Romita Choudhury, Kyle McCallum, Breda Eubank, Jason Martyn, Mel Slomp, Jason Werle, Jill Robert, Ania Kania-Richmond in Health Services Insights
