# Supplementary material for: Perceived Impacts of Health Care System Navigation on Health and Well-Being Among Patients With Musculoskeletal Conditions: A Qualitative Study
Source: Health Serv Insights. 2026 Aug 3;19:11786329261475438. doi: 10.1177/11786329261475438 (PMC13434879; doi:10.1177/11786329261475438)
Supplement: Supplementary file 2 [file sj-pdf-2-his-10.1177_11786329261475438.pdf]

Interview Guide – Patient

Condition Overview

1. **Today we are discussing shoulder, knee and low back issues. Which of these issues have you been facing?**

2. How long have you been dealing with (shoulder/knee/low back) issues?
    - Probe: Tell me how this (these) issue came about, or what brought them on?

3. Can you tell me about your experience of living with and managing your (shoulder/knee/low back) issues?
    - Probes:
        - How have these issues affected your life? Your quality of life?
        - In what ways are these issues limiting the things you're able to do?
        - How has it (the condition) impacted your life?

Care Journey

4. Tell me about your journey in getting (health) care (services) for your shoulder, knee or low back condition.
    - Probes:
        - What providers did you see? What services did you receive? Did you see the providers you needed to see, when you needed to?
        - How did you find out or decide which providers or services you should be seeing? Who made these recommendations or decisions?
        - Did you receive any diagnostic imaging (x-rays, ultrasound, MRI, CT)? Did you get the imaging you expected to receive? Did the DI help identify what care or treatment you needed?

5. Have any providers or services been difficult for you to access or receive? In what way?
    - Probes:
        - Were any referrals difficult for you to get?
        - Were any services delayed or difficult for you to access? In what way?

6. Did you pay (out-of-pocket) for any services or providers?
    - Probes:
        - How much did you pay?
        - What was the impact of paying directly?

7. Looking back over your care journey, would you say you received the care, advice and information you needed and wanted?
    - Probes:
        - Did you get the right care?
        - Were your expectations met?
        - Were you satisfied with the care you received? In what way?
        - Are there one or two words that would describe your journey?

        - Did you get advice, guidance or support to manage your condition on your own?
        - What was most helpful in your care journey?
        - What was most beneficial in your care journey?

8. Overall, what has been the most positive or memorable aspect of receiving care, and why?

9. Overall, what has been the least positive aspect of your care, and why?

Recommendations:

10. Do you have any suggestions on how the health care system can be improved to better help/support people with conditions similar to yours?
    - Probes:
        - What would have made the system better for you?
        - What would have reduced some of the barriers you faced?

11. Before we conclude, is there anything else you would like to add or share regarding your experience with the health care system to address your knee/shoulder/low back issue(s)?
